# Natural infection of filarioid nematodes in mosquitoes: An approach to neglected disease xenosurveillance and prevention in intercontinental human transit areas in Darién

**DOI:** 10.1371/journal.pntd.0013395

**Published:** 2025-09-03

**Authors:** Eddier Rivera, Anyi Tuñon, Mileyka Santos, Luisa Collado-Mariscal, Marlin González, Anayansi Valderrama

**Affiliations:** 1 Parasitological Sciences, Faculty of Natural Sciences, Exact Sciences and Technology, University of Panama, Panama City, Panama; 2 Department of Research Medical Entomology, Gorgas Memorial Institute of Health Studies, Panama City, Panama; 3 Department of Genetics and Molecular Biology, University of Panama, Panama City, Panama; University of Wisconsin-Madison, UNITED STATES OF AMERICA

## Abstract

**Background:**

Filarioid nematodes are significant vector-borne parasites affecting both humans and animals. Despite their importance, their distribution, ecological dynamics, and health implications remain poorly characterized in the Neotropics. This knowledge gap is particularly critical in high-risk areas like the Darién, a vital migratory corridor connecting the diverse ecosystems of South and Central America, where unregulated migration intersects with complex ecological and social dynamics, creating optimal conditions for the emergence and spread of filarial infections.

**Methodology/Principal findings:**

**Ethics approval:** This project was approved by the Bioethics Research Committee of Institute, Gorgas Memorial Institute for Health Studies (Approval Number: 073/CBI/ICGES/21).

Mosquito sampling was conducted across four high-mobility localities in Darién Province, Panama (Metetí, San Vicente, El Real de Santa María, and Lajas Blancas), during five collection periods, yielding 2,331 specimens representing 57 species and 10 genera. The highest species richness was recorded in El Real (S = 39) and Metetí (S = 38). In an entomological surveillance conducted in Darién, Panama, mosquitoes were collected and molecularly screened for filarial DNA, revealing widespread parasite circulation with 29 out of 57 mosquito species found positive. Infection rates calculated for individual mosquitoes showed an overall rate of 12.0% (MLE of 18.7 per 1,000 in pools), exhibiting significant geographical heterogeneity and notably elevated rates in Uranotaenia species. Positive samples underwent COX1 gene sequencing and BLAST analysis, identifying a diverse range of filarial species, including *Dirofilaria sp. ‘hongkongensis’, Brugia malayi, Onchocerca skrjabini, Setaria cervi, Onchocerca lienalis, Dirofilaria repens, and Wuchereria bancrofti*; among these, six unique samples were identified with high confidence. The majority of positive mosquitoes (75.6%) were unfed, suggesting they had not recently blood-fed from a host.

**Conclusions/Significance:**

Our findings demonstrate the significant and widespread circulation of filarial parasites across diverse mosquito species in Darién, Panama. The identification of a broad range of filarial species, notably including Brugia-like species, highlights the complex dynamic of filarial parasite circulation in this region. The consistently elevated infection rates in Uranotaenia species, among others, underscore their potential critical role as vectors. This study provides essential entomological data, emphasizing the urgent need for continuous surveillance and targeted public health interventions to mitigate filarial transmission risks.

## Author summary

Filarial worms are parasites transmitted by mosquitoes that can cause serious diseases in both people and animals. Despite their importance, we don’t fully understand where they are found or how they spread, especially in regions like Central and South America. The Darién region of Panama is a critical area because it’s a natural corridor for migration, where human movement and a rich ecosystem could create ideal conditions for these infections to emerge and spread.

We collected over 2,300 mosquitoes from four active localities in Darién, Panama. We tested these mosquitoes for the presence of filarial parasite DNA. Our screening revealed that nearly half of the mosquito species (29 out of 57) carried filarial parasites. The overall infection rate was high, with about 12% of individual mosquitoes testing positive. We also observed significant differences in infection rates between localities, and found particularly high rates in Uranotaenia mosquito species. By analyzing the parasite’s DNA, we identified a diverse range of filarial species, including Dirofilaria sp. ‘hongkongensis’, Brugia malayi, Onchocerca skrjabini, Setaria cervi, Onchocerca lienalis, Dirofilaria repens, and Wuchereria bancrofti. Importantly, six unique parasite identifications were made with high confidence. Most of the infected mosquitoes had not recently fed on blood.

Our study shows that filarial parasites are widely circulating across many different mosquito species in Darién, Panama. The presence of a variety of filarial types, including those similar to Brugia species, indicates a complex picture of how these parasites are transmitted in the region. Mosquitoes like Uranotaenia appear to play a significant role in carrying these parasites. This research provides crucial information that highlights the urgent need for ongoing monitoring of mosquitoes and targeted public health actions to reduce the risk of filarial transmission in Panama.

## Introduction

Filarioid nematodes are among the most debilitating agents of neglected diseases, disproportionately impacting vulnerable populations in tropical and subtropical regions. These parasites encompass a broad spectrum of species, including those responsible for filariasis in humans and zoonotic infections that bridge domestic animals and wildlife. Lymphatic filariasis (LF), caused primarily by *Wuchereria bancrofti*, *Brugia malayi*, and *Brugia timori*, exemplifies this burden. Manifesting as chronic lymphedema and hydrocele, LF imposes severe physical suffering, social stigma, and economic hardship, perpetuating cycles of poverty and inequality in endemic communities [1].

More than 657 million people in 39 countries remain at risk of LF despite global elimination efforts. The persistence of mosquito vectors underscores their critical role in sustaining LF transmission and other underreported filarial cycles [2]. Zoonotic filarial nematodes, such as *Dirofilaria immitis* and *Dirofilaria repens*, introduce an additional layer of epidemiological complexity by adapting to diverse hosts and vectors, mediating interactions between human and animal populations through shared mosquito vectors and overlapping ecosystems [3]. These dynamics significantly impact global health systems, presenting ecological, veterinary, and public health challenges beyond current estimates [4].

For example, *D. repens* has been associated with subcutaneous and ocular dirofilariasis in humans, as documented in Sri Lanka and other endemic regions, emphasizing its clinical importance [5]. Meanwhile, new zoonotic nematodes, such as *Brugia spp*., further illustrate the dynamic and underreported nature of filarial zoonoses [6]. Although, the detection of *Brugia* spp. are rarely detected outside Asia; however, three species, *B. lepori*, *B. beaveri*, and *B. guyanensis*, have been documented in the Americas [7]. These knowledge gaps hinder the ability to assess factors driving transmission cycles and their broader impacts across endemic and newly affected regions [8–10].

Globally, over 3,500 mosquito species have been described, many of which have been implicated in filarial transmission [11]. Several of these species are also present in Panama, where they play a role in pathogen dynamics [12]. Environmental changes, including deforestation, climate variability, human migration, and urban expansion, are reshaping mosquito distributions and altering vector-host interactions, heightening the risks of pathogen introduction and establishment [13,14]. These shifts pose significant challenges to public and zoonotic health systems, particularly in biodiverse regions like Panama [15,16]. Understanding the ecological roles of these mosquito species is therefore essential to investigate their potential as vectors of filarial nematodes and to develop strategies for mitigating disease transmission [17,18].

The Darién province, situated within the Mesoamerican Biological Corridor, exemplifies the intersection of ecological diversity and anthropogenic pressures. This biodiversity hotspot serves as a critical migratory route, facilitating wildlife movement and accommodating hundreds of thousands of human migrants annually, with over 1.2 million recorded between 2020 and 2024, many originating from filariasis-endemic regions [19]. These unregulated migration flows intersect with tropical ecosystems, where their ecological and epidemiological implications are particularly evident in mosquito-borne diseases, which remain critically understudied in the Neotropics [20,21]. As a result, the complex interplay between environmental changes and migration highlights the need for focused research in this region.

Aligned with the principles of One Health, this study investigates mosquito diversity, abundance, and infection prevalence across four ecologically distinct sites along the migratory corridor: Metetí, San Vicente, El Real de Santa María, and Lajas Blancas [22,23]. By integrating molecular techniques, such as COI-based detection and phylogenetic analysis, with traditional morphological methods, it examines the role of mosquitoes as vectors of filarial parasites and explores the applicability of xenosurveillance for detecting pathogens in high-risk areas. This integrative framework aims to refine pathogen detection methodologies and adapt advanced tools for use in resource-limited settings, laying the groundwork for strategies to address the ecological and social challenges influencing pathogen transmission in tropical environments [24,25].

## Materials and methods

### Study area

#### Mosquito collections in the Darién region.

**Fig 1:**
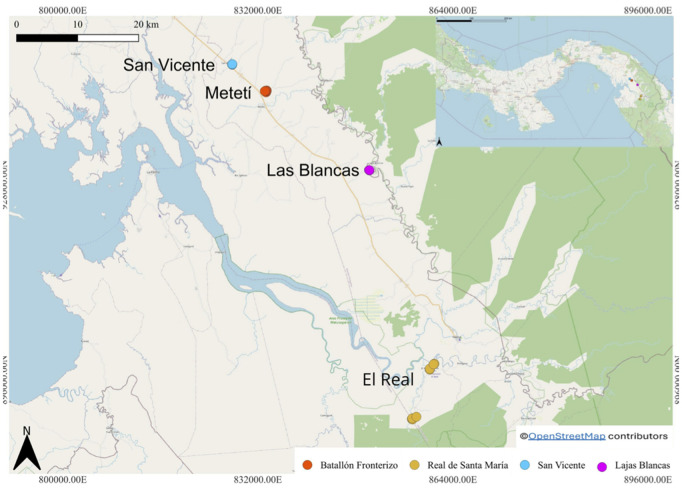
Mosquito sampling localities in Darién province. The figure illustrates the spatial distribution of sampling sites across the region. Blue and pink markers represent sites with migrant camps, whereas red and yellow markers denote sites without such camps. The map was generated by Eddier Rivera using QGIS 3.32.0 (QGIS Development Team, 2023. QGIS Geographic Information System. Open Source Geospatial Foundation Project. http://www.qgis.org/). The base map was obtained from OpenStreetMap (https://www.openstreetmap.org/#map=10/8.3433/-78.0208), which is licensed under the Open Data Commons Open Database License (ODbL) by the OpenStreetMap Foundation.

This research was conducted in Darién Province, Republic of Panama, an ecologically significant region in the country’s eastern sector. Four study sites were selected via stratified sampling to represent distinct environmental and human-influenced gradients (Fig 1). Geographical coordinates were recorded using a Garmin GPS device (Model Oregon 750t) with WGS84 datum. Site characterization combined field surveys and satellite imagery analysis (Landsat 8, 30m resolution) during 2022–2023.

The study sites included urban, agricultural, and forested landscapes. Metetí (8.52370364, -77.97145600), the primary site, serves as the region’s main administrative and commercial hub. Land use analysis indicated that urban infrastructure occupies 15% of the area, primarily concentrated in the town center. The urban area is surrounded by agricultural lands (45%) dedicated to livestock and crop production. Vegetation consists of a mosaic of cultivated pastures (*Brachiaria spp*.), annual crops, and scattered secondary forest patches dominated by pioneer species such as *Cecropia spp.*, *Ochroma pyramidale*, and *Schizolobium parahyba*. Periurban areas maintain home gardens with fruit trees and ornamental plants.

San Vicente (8.567186, -78.028861), the second site, represents a transition zone with rural development where subsistence agriculture (70%) coexists alongside remnant forest patches (30%). The agricultural matrix consists of subsistence crops such as rice, corn, and cassava, which are interspersed with fallow areas and traditional agroforestry systems. Secondary forest fragments are dominated by early and intermediate successional species like *Vochysia ferruginea*, *Apeiba tibourbou,* and *Luehea seemannii*, with an understory rich in Heliconia species and plants from the *Piperaceae* family.

El Real de Santa María la Antigua (8.111988074, -77.72694237), the third site, is a historic riverine settlement located along the Tuira River. This site illustrates the interface between human settlement and natural ecosystems, combining historic urban infrastructure (30%) with well-preserved riparian forests (70%). Riparian vegetation includes characteristic species like *Prioria copaifera*, *Pterocarpus officinalis*, and *Carapa guianensis*, alongside abundant palms such as *Socratea exorrhiza* and *Oenocarpus mapora*. The understory hosts a rich diversity of hydrophilic herbs and epiphytes.

Lajas Blancas (8.404621985, -77.819657), represents a minimally disturbed area with 70% primary forest cover. This indigenous settlement and migration station features a diverse canopy that includes valuable timber species like *Dipteryx oleifera*, *Terminalia amazonia*, and *Anacardium excelsum*. The understory hosts a rich diversity of palms, aroids, and ferns typical of mature forests. Riparian zones exhibit distinctive plant communities dominated by *Montrichardia arborescens* and various species of Heliconiaceae.

The region has a tropical humid climate (Köppen: Af), with meteorological data from local weather stations indicating annual rainfall between 1,800 and 3,000 mm. Temperature and humidity measurements using Extech RHT10 USB Data Logger placed at each site, showing mean temperatures of 25.5-27°C and relative humidity ranging from 80% to 95%. Land use percentages were determined through supervised classification of satellite imagery (ArcGIS 10.8) and validated by ground-truthing surveys during the study period.

Site selection also considered sociodemographic criteria, prioritizing locations of public health relevance related to human mobility and border security operations. Lajas Blancas and San Vicente as key surveillance points due to their roles as primary entry and exit hubs for migrants traversing the Darién jungle. The First Eastern Brigade facility in Metetí, headquarters of the National Border Service (SENAFRONT), was included to assess vector-borne disease exposure risks among border personnel stationed along the Panama-Colombia border. El Real de Santa María la Antigua was chosen for its strategic position along the migratory route through Darién National Park, offering an opportunity to study mosquito-human interactions in an area of continuous human movement through natural forest environments.

Entomological collections took place between February 2021 and November 2022. Initial sampling was restricted to Lajas Blancas in February 2021 due to COVID-19 limitations. Collections in 2022 varied in frequency across sites: SENAFRONT facilities were sampled three times (April, August, and November), Lajas Blancas twice (February 2021 and November 2022), while San Vicente (August 2022) and El Real (May 2022, including collection points at Pijibasal, Rancho Frío, and Mercadeo) were each sampled once, totaling seven field expeditions.

Sampling schedules were shaped by logistical constraints and access requirements. Access to migrant camp facilities necessitated obtaining strict institutional permits, while reaching El Real posed distinct logistical hurdles, including navigating the Tuira River by boat. Most collections were scheduled strategically during the rainy season (May to December) to optimize mosquito capture success, except for the initial sampling in February 2021 and April 2022.

#### Collection, identification, and preservation of female mosquitoes.

Two types of traps were employed: resting traps [26] and CDC light traps [27]. A total of 10 CDC light traps and 10 resting traps were used per site. Each trap was georeferenced using Garmin Oregon 750T GPS equipment and randomly distributed across each collection site. CDC traps were positioned at a height of 1 meter and baited with attractants including octanol, yeast, sugar, and water. These traps were activated at sunset and left overnight, then retrieved the following morning. Resting traps, moistened with water and local plant extracts to mimic mosquito resting sites, were installed on the first sampling day and monitored twice daily (morning and afternoon).

Sampling was conducted over three consecutive 24-hour periods at each site. However, the duration was occasionally shortened due to adverse weather conditions. CDC traps operated for 12-hour nocturnal periods per night, yielding 36 hours of sampling effort per site and accumulating 108 sampling hours per weekly collection period.

Upon trap retrieval, captured mosquitoes were anesthetized with triethylamine and identified using taxonomic keys. Morphological characteristics of the females were used to identify the genera [28–31]. They were then classified according to their physiological state (unfed, fed or depleted, gravid females) and stored in 1X PBS. Samples were transported in liquid nitrogen to the Medical Entomology Research Laboratory at the Gorgas Memorial Institute of Health Studies and stored at -80°C. This rigorous process of collection, identification, and preservation ensures the integrity and quality of the samples for subsequent analyses.

#### DNA extraction.

Specimens stored in cryovials were transferred to 1.5 mL Eppendorf tubes, and 500 μL of PBS 1X with a titanium bead was added. The tubes were then placed in a tissue homogenizer (TissueLyserII, Qiagen S.A) and shaken at 29 rpm for 1 minute. Following homogenization, the tubes were centrifuged at 10,000 rpm for 10 minutes, and two 250 μL aliquots were extracted: one stored at -80°C as a backup and the other used for DNA extraction with the Quick-DNA/RNA Pathogen Miniprep Kit.

For molecular detection of filarioid nematodes, a ~ 710 base pair (bp) fragment of the mitochondrial Cytochrome C Oxidase subunit I (COI) gene was amplified using primers COIintF (5′-TGATTGGTGGTTTTGGTAA-3′) and COIintR (5′-ATAAGTACGAGTATCAATATC-3′), following the protocol described in [32]. A blood sample infected for *Dirofilaria immitis* served as a positive control, while nuclease-free water as a negative control. DNA amplification was carried out using a Biometra TRIO series thermal cycler (Analytik Jena) following the described protocol, with expected amplicon sizes ranging between 300 and 1000 bp [32]. The amplification products were resolved by electrophoresis on a 1% agarose gel, with a 100–1000 bp molecular marker (DNA Ladder, Promega, Madison, WI, USA), and visualized under a UV transilluminator (UVP BioDoc-It System). The amplified products were purified with the QIAquick Gel Extraction Kit (Qiagen), and the purified DNA was sent to Macrogen INC Korea along with the primers used in the PCR. Chromatograms generated during the sequencing of amplified COI fragments from filarioid nematodes were analyzed using Unipro UGENE version 51.0 software to ensure data quality. Sequences were labeled with the prefix “FIP” (Filaria Panama), and assigned a unique numerical identifier based on their chronological collection order.

### Statistical analysis

#### Mosquito community.

The richness, abundance, and diversity of mosquito species in the sampling areas were analyzed based on the total number of mosquito species recorded across the four sites. Species richness was estimated using the Chao 1 index, calculated with EstimateS software v11.7.0. The abundance of each mosquito species recorded across different localities was included to improve the robustness of species richness estimates. Higher Chao 1 values indicate greater species richness within the community. The Simpson Dominance Index was used to evaluate dominance at sampling points, with values near 1.0 indicating high species dominance at a given site.

#### Mosquito infection rate.

For single specimen samples, the infection rate (%) was calculated using the formula: IR% = (Number of positive pools/ Total number of pools analyzed) × 100.

For pools with multiple specimens containing more than two individuals, prevalence was estimated using Maximum Likelihood Estimation (MLE) according to the formula: [1- (1-Y/X)1/m} x 1000, where Y = number of positive pools, X = total number of pools tested, and m = average pool size. The average pool size was calculated across all tested groups.

All statistical computations were performed using R software version 4.5.0 (R Core Team, Vienna, Austria). Confidence intervals for all prevalence estimates were calculated using the exact binomial test function (binom.test) in R.

Statistical analysis. Spatial and taxonomic heterogeneity in filarial infection rates were assessed using chi-square tests for inter-locality comparisons, followed by post-hoc pairwise analyses. Differences among mosquito genera were evaluated using Kruskal-Wallis tests with Dunn’s post-hoc comparisons. Abdominal status distribution in positive specimens was analyzed using descriptive statistics. All analyses were performed at α = 0.05 significance level.

#### Molecular identification of filarial species through COX1 gene sequencing.

To evaluate the degree of similarity between the obtained sequences and known nucleotide sequences, BLAST analyses were performed using the nucleotide collection (nr/nt) database, optimized for somewhat similar sequences (blastn) from the National Center for Biotechnology Information [33]. The resulting alignments were assessed based on three main parameters: percentage identity, query coverage, and E-value.

For the interpretation of BLAST results and the assignment of a confidence level to each identification, a composite qualitative classification system was applied as follows:

- Identity was classified as very high (≥98%), high (95–97.9%), moderate (90–94.9%), or low (<90%).
- Query coverage was classified as very high (≥95%), high (80–94.9%), moderate (50–79.9%), or low (<50%).
- E-values were interpreted as very high significance (≤ 1 × 10 ⁻ ⁵⁰), high significance (> 1 × 10 ⁻ ⁵⁰ to ≤ 1 × 10 ⁻ ²⁰), moderate significance (> 1 × 10 ⁻ ²⁰ to ≤ 1 × 10 ⁻ ⁵), and not significant (> 1 × 10 ⁻ ⁵).

Sequences were subsequently categorized into three general confidence levels for species identification, based on a combined assessment of these parameters:

- High-confidence identifications: Assigned to sequences exhibiting very high E-value significance (≤ 1 × 10 ⁻ ⁵⁰)and very high (≥95%) or high (80–94.9%) query coverage, regardless of the percentage identity (which could fall into the moderate or even low range). This indicates an extensive and highly specific alignment.
- Moderate-confidence identifications: Assigned when sequences showed a percentage identity between 90% and 97.9% (moderate to high), with moderate query coverage (50–79.9%), and high or moderate E-value significance. This category also included identifications with very high identity and E-value but with query coverage that, while not low, did not meet the criteria for “high-confidence.”
- Low-confidence identifications: Primarily assigned when query coverage was low (<50%), despite a high percentage identity, or when all three parameters collectively indicated weak similarity or a partial alignment, suggesting a less robust match.

Ethics approval: This project was approved by the Bioethics Research Committee of Institute, Gorgas Memorial Institute for Health Studies (Approval Number: 073/CBI/ICGES/21).

## Results

### Mosquito community

A total of 2,331 mosquitoes were collected across four sampling zones in Darién Province, representing 10 genera and 57 species. The most abundant genera were *Culex* (20%), *Mansonia* (17%), *Anopheles* (14%), and *Aedes* (6.8%), with several species recognized as vectors of filarioid nematodes affecting humans and animals worldwide. Additionally, other genera included *Psorophora* (17%), *Coquillettidia* (4.4%), and *Uranotaenia* (6.9%). Less frequent genera, such as *Trichoprosopon* (0.09%) and *Wyeomyia* (0.04%), were also recorded. Among the collected individuals, most were captured in the area of Metetí (40%), followed by Real de Santa Maria (37.4%), Lajas Blancas (18.9%), and San Vicente (3.5%). The most abundant species were *Mansonia titillans* (14.2%), *Anopheles albimanus* (12.0%), and *Psorophora cingulata* (8.5%).

The analysis of mosquito diversity revealed contrasting patterns among the four studied localities. El Real and Metetí exhibited the highest species richness (S = 39 and 38 species, respectively) and total abundance (873 and 933 individuals), followed by Lajas Blancas (S = 25, abundance = 442) and San Vicente, which showed the lowest values (S = 16, abundance = 83). Simpson’s diversity index (1-D) indicated high diversity across all localities, with the highest values observed in Metetí (0.9055) and San Vicente (0.9013), followed by El Real (0.872) and Lajas Blancas (0.6926). The Chao-1 estimator suggested that the sampling effort was adequate, as the estimated richness values (43.99, 30.24, 18.96, and 39.2 for El Real, Lajas Blancas, San Vicente, and Metetí, respectively) were relatively close to the observed values, indicating that a significant proportion of the expected diversity was captured at each locality (Table 1.)

**Table 1:** Comparative analysis of mosquito community structure and diversity metrics in four localities of Darién Province, Panama.

Specie	El Real	Lajas Blancas	San Vicente	Metetí
*Aedeomyia squamipennis*	1	20	0	65
*Aedes albopictus*	2	0	0	0
*Aedes eupoclamus*	143	0	0	0
*Aedes scapularis*	0	0	0	2
*Aedes serratus*	2	0	0	4
*Aedes sp.*	3	0	0	0
*Aedes taeniorhynchus*	3	0	0	0
*Aedes zavortinki*	0	0	0	1
*Anopheles albimanus*	7	221	3	49
*Anopheles malefactor*	0	7	0	10
*Anopheles neomaculipalpus*	6	1	0	4
*Anopheles punctimacula*	1	3	0	0
*Anopheles sp.*	2	0	0	4
*Anopheles triannulatus*	0	2	0	0
*Coquillettidia sp.*	6	0	0	0
*Coquillettidia venezuelensis*	56	6	1	34
*Culex iolambdis*	0	1	0	3
*Culex adamesi*	1	0	0	0
*Culex amazonensis*	6	1	0	1
*Culex conservator*	1	0	0	1
*Culex corniger*	0	1	0	0
*Culex coronator*	20	5	8	68
*Culex crybda*	0	0	1	0
*Culex (Culex) sp.*	15	1	0	18
*Culex declarator*	0	1	3	13
*Culex dunni*	2	0	4	15
*Culex erraticus*	0	1	0	0
*Culex* grupo *intrincatus*	0	0	2	2
*Culex* grupo *pilosus*	1	0	0	8
*Culex interrogator*	12	9	15	41
*Culex inhibitator*	0	0	4	11
*Culex (Melanoconion) sp.*	1	4	0	3
*Culex nigripalpus*	24	2	6	97
*Culex ocossa*	2	19	0	4
*Culex pedroi*	8	0	0	27
*Culex lactator*	0	0	1	0
*Culex quinquefasciatus*	78	0	0	7
*Culex spissipes*	8	4	1	5
*Culex theobaldi*	4	6	4	43
*Culex vomerifer*	43	0	0	0
*Mansonia dyari*	0	0	0	23
*Mansonia humeralis*	0	0	0	49
*Mansonia indubitans*	2	0	0	0
*Mansonia titillans*	2	101	4	224
*Psorophora albipes*	1	0	0	0
*Psorophora cilipes*	1	0	0	0
*Psorophora cingulata*	169	3	12	16
*Psorophora confinnis*	185	0	0	0
*Psorophora ferox*	2	0	0	3
*Psorophora sp.*	0	0	0	4
*Trichoprosopon digitatum*	0	0	0	2
*Uranotaenia apicalis*	3	2	0	27
*Uranotaenia hystera*	1	6	0	0
*Uranotaenia leucoptera*	0	0	0	2
*Uranotaenia lowii*	48	15	14	42
*Uranotaenia nataliae*	1	0	0	0
*Wyeomyia arborea*	0	0	0	1
SAMPLING METRICS				
Species Richness (S)	39	25	16	38
Total Abundance	873	442	83	933
Simpson’s Diversity Index (1-D)	0.872	0.6926	0.9013	0.9055
Chao-1 Index	43.99	30.24	18.96	39.2

### Mosquito infection rate

We analyzed a total of 2,331 mosquitoes (750 pools, 430 individuals), identifying 147 positive for filariae across 67 tubes/pools (50 individuals, 17 pooled). Conversely, 684 pools (2,184 mosquitoes) were negative. Among 10 mosquito genera (54 species) analyzed, 7 tested positive for filarial DNA, while 3 were negative.

The analysis of infection rate and Maximum Likelihood Estimation data revealed significant patterns among the mosquito species studied. Overall, 57 species were analyzed, and 29 species (50.9%) tested positive for filarial infection.

**Infection rate analysis for individual mosquito specimens:** Natural filarial infection was detected across all four sampling localities, with an overall infection rate of 12.0% (95% CI: 8.6%-14.7%) among individual mosquitoes examined. Locality-specific infection rates varied considerably, ranging from 6.0% (95% CI: 3.0%-13.2%) in Lajas Blancas to 17.0% (95% CI: 3.6%-39.3%) in both San Vicente and Metetí, with El Real showing an intermediate rate of 9.0% (95% CI: 5.3%-14.2%). The highest infection rates were observed in *Uranotaenia* species, with *U. apicalis* demonstrating 100% positivity (95% CI: 16.0%-100.0%) in Metetí and *U. lowii* showing perfect detection rates in San Vicente (100%; 95% CI: 3.0%-100.0%) despite smaller sample sizes. *Aedeomyia squamipennis* exhibited the most robust infection signal with 30.0% positivity (95% CI: 13.0%-53.0%) in Metetí, representing the highest lower-bound confidence interval (13.0%) across all species-locality combinations and involving the largest number of infected pools (7/23). Other notable species included *Coquillettidia venezuelensis* in Lajas Blancas (50.0%; 95% CI: 12.0%-88.0%) and *Aedes eupoclamus* in El Real (13.0%; 95% CI: 2.0%-40.0%), which maintained significant detection rates despite larger sampling efforts. Multiple *Culex*, *Psorophora*, and *Mansonia* species demonstrated consistent infection rates of 33.0% (95% CI: 7.0%-70.0%) in Metetí, indicating broad filarial circulation across diverse mosquito taxa within this locality (Table 2).

**Table 2:** Mosquito Species Composition and Infection Rates by Collection Locality.

Species	Positive individuals	Total individuals	Infection Rate (%)	95% CI Lower	95% CI Upper
El Real					
*Culex (Melanoconion) sp.*	1	1	100.0	3.0	100.0
*Culex conservator*	1	1	100.0	3.0	100.0
Aedes albopictus*	1	2	50.0	1.0	99.0
*Culex dunni*	1	2	50.0	1.0	99.0
Psorophora ferox**	1	2	50.0	1.0	99.0
*Uranotaenia lowii*	2	4	50.0	7.0	93.0
*Aedes sp.*	1	3	33.0	1.0	91.0
Culex interrogator***	1	6	17.0	0.0	64.0
Culex pedroi***	1	6	17.0	0.0	64.0
*Aedes eupoclamus*	2	15	13.0	2.0	40.0
Culex nigripalpus***	1	14	7.0	0.0	34.0
Psorophora confinnis**	1	15	7.0	0.0	32.0
*Lajas Blancas*					
Culex erraticus***	1	1	100.0	3.0	100.0
Coquillettidia venezuelensis***	3	6	50.0	12.0	88.0
*Uranotaenia apicalis*	1	2	50.0	1.0	99.0
*Aedeomyia squamipennis+*	1	5	20.0	1.0	72.0
*San Vicente*					
Culex declarator***	1	1	100.0	3.0	100.0
*Uranotaenia lowii+*	1	1	100.0	3.0	100.0
*Culex interrogator*	1	3	33.0	1.0	91.0
*Metetí*					
*Uranotaenia apicalis+*	2	2	100.0	16.0	100.0
Culex ocossa***	1	2	50.0	1.0	99.0
Culex coronator***	3	9	33.0	7.0	70.0
Mansonia humeralis***	1	3	33.0	1.0	91.0
Psorophora cingulata**	3	9	33.0	7.0	70.0
*Uranotaenia lowii+*	3	9	33.0	7.0	70.0
*Aedeomyia squamipennis+*	7	23	30.0	13.0	53.0
*Culex dunni*	1	5	20.0	1.0	72.0
Culex quinquefasciatus*	1	5	20.0	1.0	72.0
Mansonia titillans***	2	11	18.0	2.0	52.0
*Culex (Culex) sp.*	1	7	14.0	0.0	58.0
*Coquillettidia venezuelensis*	1	9	11.0	0.0	48.0
*Culex theobaldi*	1	9	11.0	0.0	48.0

CI = Confidence Interval. Only mosquito species with Filaria-positive. Infection rates are calculated as the proportion of positive pools out of total pools tested for each species-locality combination. *Species incriminated in arbovirus transmission in urban areas.**anthropophilic, especially in or near forested and livestock areas.***Feed on humans opportunistically. + Primarily ornithophilic.

**Maximum Likelihood Estimation (MLE) analysis for pools with multiple mosquitoes** revealed filarial circulation across mosquito populations, with an overall estimated prevalence of 18.7 per 1,000 individuals (95% CI: 12.3-28.5 per 1,000) based on examination of pooled mosquitoes across four collection localities. Locality-specific MLE values demonstrated considerable heterogeneity, ranging from 55.6 per 1,000 in Lajas Blancas (95% CI: 1.4-195.1 per 1,000) to 96.4 per 1,000 in San Vicente (95% CI: 2.1-285.5 per 1,000), with intermediate values observed in Metetí (24.8 per 1,000; 95% CI: 8.9-68.7 per 1,000) and El Real (23.4 per 1,000; 95% CI: 11.8-46.3 per 1,000). The highest individual species MLE was recorded in *Uranotaenia leucoptera* from Metetí (1,000 per 1,000; 95% CI: 0.986-∞), followed by *Culex spissipes* from El Real (179.7 per 1,000; 95% CI: 3.6-1,000 per 1,000), though both estimates reflect small sample sizes with wide confidence intervals. More robust estimates with substantial sample sizes included *Culex vomerifer* (101.1 per 1,000; 95% CI: 14.1-220.0 per 1,000) and *Uranotaenia lowii* (79.6 per 1,000; 95% CI: 13.1-526.5 per 1,000) from El Real, and *Psorophora cingulata* from San Vicente (96.4 per 1,000; 95% CI: 2.1-285.5 per 1,000). Notably, *Uranotaenia lowii* demonstrated consistent filarial presence across multiple localities, with detections in both El Real and Metetí, indicating this species’ widespread involvement in filarial transmission cycles. The genus *Uranotaenia* showed particularly high infection estimates across all collection sites where present, with MLE values consistently exceeding 30 per 1,000 individuals (Table 3).

**Table 3:** Mosquito Species Composition and Infection Rates by Collection Locality.

Species	Positive Pools	Total Mosquitoes	MLE (per 1,000)	95% CI Lower	95% CI Upper
*El Real*					
*Aedes eupoclamnus*	1	128	8.0	0.10	52.1
*Coquillettidia venezuelensis*	2	49	45.1	4.1	257.8
Culex spissipes***	1	7	179.7	3.6	1000.0
Culex vomerifer***	3	43	101.1	14.1	220.0
*Uranotaenia lowii*	3	44	79.6	13.1	526.5
*Lajas Blancas*					
Culex ocossa***	1	18	61.9	1.4	195.1
*San Vicente*					
*Psorophora cingulata*	1	12	96.30	2.1	285.5
*Metetí*					
*Aedeomyia squamipennis*	1	42	25.1	0.6	74.5
*Mansonia titillans*	1	213	4.8	0.1	29.3
*Uranotaenia apicalis*	1	25	47.5	1.0	131.6
*Uranotaenia leucoptera*	1	2	1000	0.9	NAN
*Uranotaenia lowii*	1	33	32.6	0.8	99.9

CI = Confidence Interval. Only mosquito species with Filaria-positive pools. MLE (per 1,000)MLE (per 1,000): Maximum Likelihood Estimate of infection rate per 1,000 mosquitoes, calculated from pooled data. ***Feed on humans opportunistically.

Statistical comparisons revealed significant heterogeneity in filarial infection rates across both geographical and taxonomic dimensions. Inter-locality analysis demonstrated significant variation in infection prevalence (χ² = 14.3, df = 3, p < 0.05), with post-hoc pairwise comparisons identifying Meteti and San Vicente as high-transmission foci compared to El Real and Lajas Blancas (p < 0.01). At the taxonomic level, Kruskal-Wallis analysis revealed significant differences among mosquito genera (H = 8.21, df = 6, p < 0.05), with Uranotaenia species showing consistently elevated infection rates compared to other genera (Dunn’s test, p < 0.01).

Analysis of the abdominal status distribution among positive specimens (n = 123) revealed that the majority were unfed (75.6%, n = 93), followed by gravid females (17.1%, n = 21). Blood-fed and depleted specimens comprised a smaller proportion of the sample, representing 4.9% (n = 6) and 2.4% (n = 3), respectively.

### Molecular identification of filarial species through COX1 gene sequencing

COX1 gene sequencing and subsequent BLAST analysis were performed on 12 filarial samples, yielding sequence lengths ranging from 619 to 736 base pairs. The majority of samples (8/12) produced sequences ≥670 bp.

Based on the predefined criteria for sequence identity, query coverage, and E-value (as detailed in Methods and presented in Table 4), identifications were categorized into three confidence levels.

**Table 4:** Molecular Identification of Filarial COX1 Gene Sequences Detected in Mosquito Samples from Darién, Panama.

No	Sample ID	Top Hit Scientific Name	Identity (%)	Query Coverage (%)	E-value	Significance	NCBI Accession
1	FIP59	*Onchocerca skrjabini*	85.39%	95%	0.0	Very high	ON854653.1
2	FIP60	*Onchocerca lienalis*	81.12%	78%	1,00E-68	Very high	KX853325.1
3	FIP61	*Setaria cervi*	94.33%	25%	1,00E-51	Very high	MK360913.1
4	FIP62	*Setaria cervi*	94.20%	24%	1,00E-48	Hight	MK360913.1
5	FIP63	*Onchocerca llenalis*	87.37%	42%	7,00E-81	Very high	KX853326.1
6	FIP64	*Dirofilaria sp. ‘hongkongensis’*	89.25%	100%	0.0	Very high	OR616713.1
7	FIP77	*Brugia malayi*	88.68%	99%	0.0	Very high	MN564741.1
8	FIP79	*Brugia malayi*	87.93%	99%	0.0	Very high	MN564741.1
9	FIP80	*Brugia malayi*	88.84%	99%	0.0	Very high	MN564741.1
10	FIP81	*Brugia malayi*	86.75%	98%	0.0	Very high	MN564741.1
11	FIP87	*Sertaria cervi*	92.91%	31%	4,00E-47	High	MK360913.1
12	FIP07	*Dirofilaria repens*	97.73%	9%	3,00E-08	Moderate	PQ560501.1
12	FIP07	*Wuchereria bancrofti*	100%	9%	2,00E-10	Moderate	KY883763.1

Molecular identification of filarial sequences was performed by BLAST analysis against the NCBI nucleotide collection (nr/nt) database. Best hits are presented based on their percentage identity, query coverage, and E-value. **No.:** Sequential sample number. **Sample ID:** Unique identifier for each mosquito sample/pool from which filarial DNA was detected. **Top Hit Scientific Name:** The scientific name of the best-matching sequence in the database. Species names are italicized. **Identity (%):** Percentage of identical nucleotides between the query sequence and the top hit. **Query Coverage (%):**Percentage of the query sequence that is covered by the alignment to the top hit. **E-value:** The expected number of random matches for the given alignment. Lower E-values indicate higher significance. **Significance:** Qualitative interpretation of the BLAST hit strength, categorized as ‘Very high’, ‘High’, or ‘Moderate’, based on a composite assessment of percentage identity, query coverage, and E-value criteria as detailed in the Methods section. **GenBank Accession No.:** NCBI GenBank accession number of the best-matching sequence.

High-confidence identifications (n = 6) included sample FIP64 matching *Dirofilaria sp*. ‘hongkongensis’ (89.25% identity, 100% query coverage, E-value 0.0), and samples FIP77, FIP79, FIP80, and FIP81 matching *Brugia malayi* (86.75-88.84% identity, 98–99% query coverage, E-value 0.0). Additionally, sample FIP59 matched *Onchocerca skrjabini* (85.39% identity, 95% query coverage, E-value 0.0). In all these cases, the very high E-values and high to very high query coverage supported a robust species identification despite varying identity percentages.

Moderate-confidence identification (n = 1) comprised sample FIP60 matching *Onchocerca lienalis* (81.12% identity, 78% query coverage, E-value 1.00E-68). This classification reflects its moderate query coverage, which, while substantial, did not meet the criteria for a high-confidence match.

Low-confidence identifications (n = 6) primarily due to low query coverage, included three samples (FIP61, FIP62, FIP87) matching *Setaria cervi* (92.91-94.33% identity, but 24–31% query coverage), and sample FIP63 matching *Onchocerca lienalis* (87.37% identity, 42% query coverage). Sample FIP07 also fell into this category, with matches to both *Dirofilaria repens* (97.73% identity) and *Wuchereria bancrofti* (100% identity), both showing critically low query coverage (9%).

Definitive species identification was achieved for 6 of 12 unique samples (50%) at a high-confidence level. The remaining samples (one moderate-confidence, five low-confidence unique samples, plus the dual low-confidence match for FIP07) require additional molecular approaches for more conclusive taxonomic determination.

## Discussion

The mosquito species composition in Darién reflects patterns characteristic of highly biodiverse Neotropical regions, with *Culex*, *Mansonia*, and *Anopheles* genera dominating these communities. This dominance aligns with previous studies conducted in ecological transition zones where these genera thrive due to their adaptability to diverse microhabitats [34,35], and similar patterns emerge in the Brazilian Amazon in areas where forest and anthropogenic environments converge [34].

Our diversity indices, including Simpson and Chao-1 values recorded at sites such as El Real and Metetí, demonstrate a community structure consistent with other Neotropical regions exhibiting high vector diversity [36]. These indices reveal well-structured communities characterized by high species richness and equitable abundance distribution, features typical of transitional ecosystems that provide multiple ecological niches [36].

The presence of *Culex*, *Mansonia*, and *Anopheles* species in Darién carries significant epidemiological implications due to their documented roles as filariasis disease vectors in other tropical regions. Specifically, *Culex quinquefasciatus* serves as the primary vector of *Wuchereria bancrofti* in urban areas of Brazil, playing a crucial role in lymphatic filariasis transmission [37–39]. Given its prevalence in urban environments and anthropophilic behavior, experimental evaluation of local *C. quinquefasciatus* populations for filarial parasite vectorial competence is warranted [40,41].

*Mansonia* species have demonstrated vector competence for *Brugia malayi* in Southeast Asia, with research indicating higher infection rates for species like *M. longipalpis* than *M. annulata* [42–44]. However, evolutionary proximity does not guarantee equivalent transmission capabilities across different geographical regions [45]. Similarly, *Anopheles* species, globally recognized as malaria vectors, have also demonstrated competence in *Brugia* transmission in Asian contexts [46,47]. Given that vector competence can vary significantly even among species within the same genus, rigorous experimental assessment of *Mansonia* and *Anopheles* species present in Darién is essential and region-specific evaluations are important [48].

**Fig 2:**
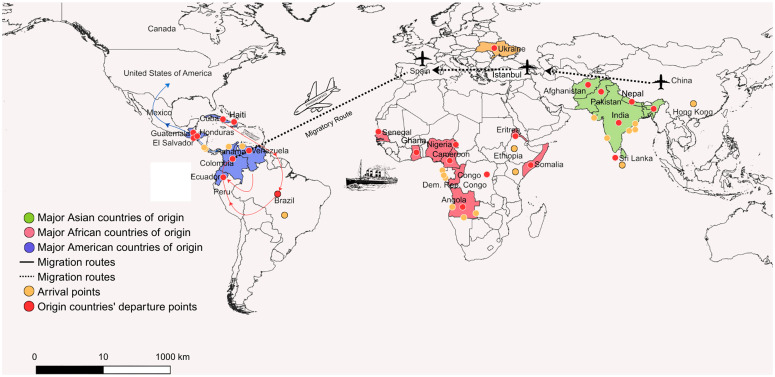
Map of Transcontinental Migration Routes through the Darién Gap. This map illustrates key international migration routes crossing the Darién Gap, emphasizing the primary countries of origin and critical arrival points. Regions are color-coded to indicate major migrant origins: Asia is represented by green color, Africa by orange-red color, and the Americas by blue. Arrival points are marked with orange circles, departure point with red circles, while whole and dashed lines depict the migration paths. Produced by Panama’s National Border Service (SENAFRONT), the map serves as a strategic tool to contextualize the complex dynamics of human mobility across continents. The map was generated by Anayansi Valderrama using QGIS 3.32.0 (QGIS Development Team, 2023. QGIS Geographic Information System. Open Source Geospatial Foundation Project. http://www.qgis.org/). The base map was obtained from Natural Earth (https://www.naturalearthdata.com/downloads/10m-cultural-vectors/10m-admin-0-countries/), which is in the public domain.

This ecological complexity is particularly significant given Darién’s role as a unique biogeographical and migratory bottleneck. As illustrated in (Fig 2), this region serves as a critical pathway for diverse international migration, with origins spanning continents and clear arrival points. Research consistently highlights this region’s critical importance in broader pathogen dynamics, including yellow fever, leishmaniasis, and expanding human filarial infections, due to constant interactions between humans, local fauna, and vectors within an environment of significant sanitary vulnerability [49–52]. This context underscores Darién as an ideal setting for robust entomological surveillance and research into complex vector-pathogen-host interactions.

Our molecular analysis revealed remarkably high filarial DNA detection rates in Darién’s mosquito populations, ranging from 12.0-18% of pools, a prevalence that exceeds those reported from some established endemic regions such as Tanzania (1.7%) and India (6.66%) [53,54]. Filarial DNA was detected across an exceptionally broad taxonomic distribution, with 29 species testing positive, suggesting widespread circulation beyond typically recognized specificities [55].

**Fig 3:**
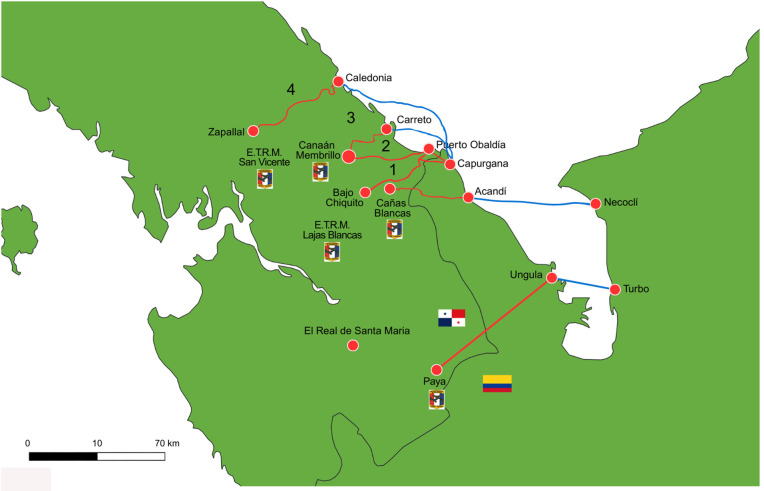
Map of Irregular Migration Routes between Colombia and Panama. Informal migration routes through the Darién Gap, highlighting primary pathways, transit points, and migrant stations. Geographic features such as protected areas and Indigenous territories are marked to illustrate factors influencing migration patterns. Migrant stations (ETRM San Vicente and ETRM Lajas Blancas) are noted for their dual role in providing humanitarian assistance and serving as data collection sites. Produced by Panama’s National Border Service (SENAFRONT). The map was generated by Anayansi Valderrama using QGIS 3.32.0 (QGIS Development Team, 2023. QGIS Geographic Information System. Open Source Geospatial Foundation Project. http://www.qgis.org/). The base map was obtained from Natural Earth (https://www.naturalearthdata.com/downloads/10m-cultural-vectors/10m-admin-0-countries/), which is in the public domain.

This high prevalence and broad diversity underscore favorable ecological conditions for filaria circulation, emphasizing the urgent need for comprehensive, localized vector competence studies. Geographic analysis further revealed significant spatial heterogeneity in detection rates, with Metetí, San Vicente, Lajas Blancas, and El Real showing differential patterns, likely reflecting localized ecological factors and increased host-vector interactions, especially along migratory routes. The irregular migration routes between Colombia and Panama, including primary pathways, transit points, and key migrant stations like San Vicente and Lajas Blancas, are detailed in (Fig 3). These stations play a dual role in providing humanitarian assistance and serving as data collection sites, and geographic features such as protected areas and Indigenous territories are known to influence migration patterns [56–62].

Notably, filarial DNA was detected in *Uranotaenia* species (*U. apicalis*, *U. lowii*, and *U. leucoptera*), which is a significant finding as this genus is generally recognized as specialized, non-anthropophilic, and primarily associated with avian malaria [63]. The high MLE for *U. lowii* (79.6 per 1000) further suggests a potential, previously unrecognized role in filaria biology in Darién. Similarly, *Aedeomyia squamipennis* presented a notable 30.0% filarial DNA detection rate. Given its predominantly ornithophilic feeding, this finding warrants investigation into its role as a potential bridge vector in areas of animal, human-bird contact [64].

We also repeatedly detected filarial DNA in highly anthropophilic and medically important species such as *Culex quinquefasciatus* (a primary *Wuchereria bancrofti* vector in urban areas of Brazil [38,39]), *Culex vomerifer* (incriminated in arbovirus transmission in Panama [65–67]), and *Psorophora cingulata* (highly anthropophilic, associated with zoonotic arboviruses like VEEV [68,69]). These detections highlight their strong potential as filarial vectors in Darién, emphasizing the urgent need for experimental vector competence evaluation for these local populations [40,70]. While *Mansonia* and *Anopheles* species are known *Brugia* vectors in other contexts, their variable competence necessitates rigorous local assessment [42–48,71].

Analysis of infected mosquito abdominal status showed that unfed (75.6%) and gravid (17.1%) stages were most commonly infected, aligning with periods of high host-seeking or oviposition behaviors [72]. Importantly, the absence of a direct association between mosquito abundance and filarial DNA detection rates suggests that inter-species variations reflect specific ecological and behavioral factors, rather than simple population density, pointing towards complex and potentially novel mosquito-parasite interactions related to parasite circulation and potential for exposure.

Molecular identification through COX1 sequencing revealed a diverse array of filarial species. However, its efficacy for precise species assignment was limited for some samples, underscoring the challenges of using partial mitochondrial data and the need for multigene approaches in taxonomically complex nematode groups, as high intraspecific variability can lead to taxonomic confusion [32,73–75].

Notably, *Brugia*-like sequences were identified in four independent samples, showing high sequence identities (>85% and 98% query coverage) similar to *Brugia spp.* These sequences likely correspond to American continental species such as *B. beaveri*, *B. lepori*, or *B. guyanensis*, given *B. malayi*’s geographic restriction to Southeast Asia and the documented presence of American zoonotic *Brugia* species [18,76–80].Epidemiologically, the detection of *Brugia*-like sequences in *Coquillettidia venezuelensis* and *Culex occossa* collected specifically in Lajas Blancas is highly significant [81]. This location serves as one of the primary migrant reception and transit centers in the Darién corridor [19,59], creating a unique confluence of potentially competent vectors and human migratory flows. This raises two critical hypotheses: 1) potential parasite introduction by migrants from endemic regions, or 2) existing enzootic American *Brugia* cycles encountering novel human exposure opportunities through vector-human contact. This positions Lajas Blancas as a crucial surveillance site [81,82].

Other notable detections include *Dirofilaria*-like sequences (89.25% similarity with *Dirofilaria sp. ‘hongkongensis’*) in *Culex coronator*, an opportunistic feeder. This result requires cautious interpretation, as *D. ‘hongkongensis’* is considered a *nomen nudum*, lacking formal taxonomic description and ZooBank registration [83]. This denomination was proposed primarily based on molecular data from Asian cases [84], thereby limiting its nomenclatural validity according to the International Code of Zoological Nomenclature. Consequently, *D. immitis* remains the principal etiological agent of dirofilariasis in the Americas [85,86], and our finding could indicate potential regional genetic variants or underscore COX1 marker limitations. Setaria-like DNA, consistent with *Setaria cervi* (a filarial parasite commonly associated with wild and domestic ungulates [87]), was found in *Culex coronator* and *Uranotaenia apicalis*. This suggests the potential existence of enzootic cycles involving native ungulates in Darién, and possibly greater feeding plasticity in *U. apicalis* under specific ecological conditions [88–90]. Furthermore, the unusual detection of *Onchocerca* in culicidae mosquitoes (*Mansonia titillans*, *Culex coronator*), typically vectored by black flies (*Simulium*) [91–93], prompts investigation into alternative transmission cycles. A diagnostic challenge was observed with sample FIP07 (*Culex conservator* from El Real), showing ambiguous matches for *Dirofilaria repens* and *Wuchereria bancrofti* due to critically low query coverage. This highlights the inherent limitation of partial COX1 sequences in differentiating epidemiologically distinct genera, emphasizing the need for robust molecular surveillance programs to avoid misidentification and to distinguish between zoonotic and human-specific pathogens.

## Limitations

Despite its significant contributions, this study has several limitations that warrant consideration. Firstly, methodological constraints related to sample preservation impacted downstream analyses. The requirement for liquid nitrogen preservation, due to the 273-kilometer distance to the nearest -80°C facility, likely caused cryofracture of fragile parasite structures. This precluded examination of key morphological features necessary for definitive identification of filarial parasites and prevented confirmation of their developmental stages within the mosquito vectors. Additionally, samples were pooled with up to 10 individuals for resource efficiency. While practical, this approach may have diluted low-intensity infections or rare haplotypes, potentially limiting genetic analysis resolution and masking true prevalence rates.

Secondly, molecular identification faced inherent challenges. COX1-based identification presented significant limitations due to the scarcity of filarial reference sequences in GenBank, especially for Neotropical species. This often resulted in moderate confidence identifications that require cautious interpretation, primarily due to a lack of highly homologous sequences for robust comparison [73]. As a consequence, specific species-level assignments were not always definitive (e.g., ‘*Brugia*-like’, FIP07 ambiguity), precluding precise conclusions on species without further multi-gene markers or morphological confirmation.

Most importantly, parasite DNA detection in mosquito vectors does not confirm vector competence or active transmission capacity. This requires experimental demonstration of complete parasite development and viable transmission under controlled conditions [48,94]. While our findings indicate mosquito exposure or infection, they do not establish their role as biological vectors.

**Fig 4:**
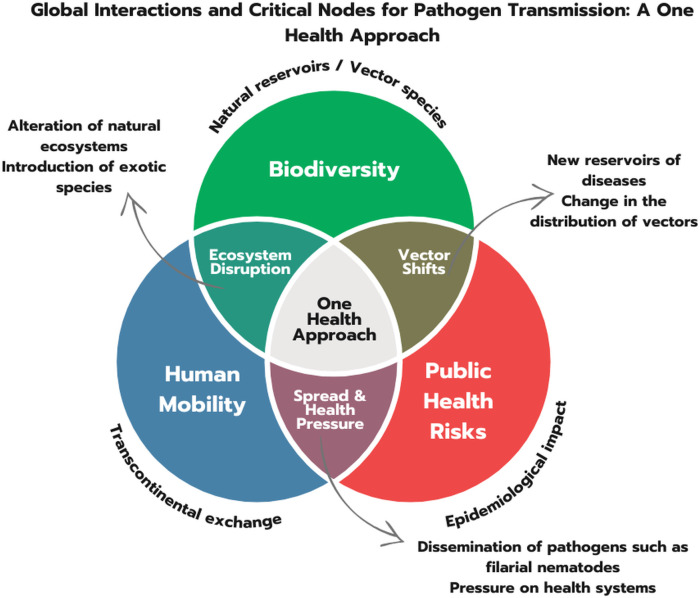
Global Interactions and Critical Nodes for Pathogen Transmission: A One Health Approach. This Venn diagram illustrates the interconnected relationships between biodiversity, human mobility, and public health risks, emphasizing the One Health approach. Overlapping regions highlight: Biodiversity & Public Health: New disease reservoirs and changes in vector distribution. Human Mobility & Public Health: Pathogen spread and increased pressures on health systems. Biodiversity & Human Mobility: Ecosystem alterations leading to vector shifts and pathogen emergence. The central overlap emphasizes the One Health approach as a framework for addressing complex global health challenges. Diagram created by Marlin González.

Considering the totality of our findings and the inherent limitations, our molecular and ecological analyses collectively underscore the critical importance of understanding pathogen transmission dynamics in Darién. This study demonstrates the inherent value of integrating molecular techniques with traditional entomological methods to significantly enhance surveillance capabilities [95–97]. By detecting filarial DNA across a diverse range of vectors, our findings vividly illustrate the complex dynamics of interconnectivity between biodiversity and public health a central principle of the “One Health” approach (Fig 4). Our work offers novel insights into intricate relationships between migration, mosquito diversity, and filarial DNA distribution in Darién. The site-specific detection patterns and identified potential vector-parasite associations provide new understanding of how localized conditions influence broader regional epidemiological dynamics.

This comprehensive and integrated surveillance strategy proves a valuable tool for identifying high-risk areas where vector diversity and zoonotic risks converge, thereby serving as a robust early warning system for resource-limited regions. This collectively reinforces the urgent need for targeted interventions for early detection and containment, underscoring the potential value of integrated strategies encompassing continuous vector monitoring, environmental management, and enhanced surveillance cooperation as essential tools to effectively address neglected tropical diseases in such complex and dynamic regions, thus preventing disproportionate public health impacts [98–101].

## Conclusion

Our molecular and ecological analyses reveal Darién as a region of critical importance for filarial disease surveillance. The most notable finding is the detection of *Brugia*-like sequences in mosquito vectors, particularly *Culex quinquefasciatus*, at Lajas Blancas—a primary migrant transit hub. This spatial association between potential lymphatic filariasis-related sequences and human migratory flows demands urgent epidemiological attention. While highly similar to *Brugia malayi*, these sequences likely represent native American *Brugia* species (*B. beaveri*, *B. lepori*, or *B. guyanensis*) potentially encountering novel human-vector interactions in migration sites.

Beyond *Brugia*, we detected four distinct filarial genera (*Brugia, Dirofilaria, Onchocerca*, and *Setaria*) with high overall detection rates, suggesting favorable ecological conditions for parasite circulation. The presence of filarial DNA in *Uranotaenia* species, previously unassociated with human filariasis, expands our understanding of transmission networks. Although DNA detection doesn’t confirm vector competence, the documented roles of *Mansonia*, *Culex*, and *Coquillettidia* as filarial vectors, combined with their presence in migration areas, merits attention.

These findings highlight critical epidemiological implications: the potential for lymphatic filariasis introduction via infected migrants; the possible activation of enzootic cycles involving American *Brugia* species to include human hosts; and the establishment of new transmission foci in this hemispheric migration corridor.

Systematic investigation is urgently needed to: 1) confirm taxonomic identity via multigenic analysis; 2) experimentally evaluate vector competence; 3) identify vertebrate reservoirs; and 4) establish enhanced surveillance protocols, including migrant population screening.

Our study demonstrates the value of integrating molecular techniques with traditional entomological methods for pathogen surveillance in migration corridors. This approach reveals patterns often undetected by conventional methods. The convergence of human migration, potentially competent vectors, and molecular evidence of filarial circulation positions Darién as a surveillance priority for understanding filarial disease dynamics in the Americas.

## Supporting information

S1 File Raw data of mosquito collection and characterization in Darien, Panama. This table provides individual mosquito data, including collection date (Year, Month, Day), locality (Lajas Blancas, El Real, San Vicente, Metetí), identified species, sex (female), number of individuals, physiological state (fed, unfed, sugar-fed, gravid), and the result (positive or negative) of filarial DNA detection via COX1 amplification.
(XLSX)

S2 File Partial Cytochrome Oxidase I (COXI) gene sequences of filariae detected in mosquitoes. This FASTA file contains the nucleotide sequences obtained from mosquito samples that tested positive for filarial infection. Each entry includes a unique mosquito sample identifier and the corresponding filarial taxonomic information when determined.
(FAS)
